# Spike-timing-dependent plasticity offers delay-gated oscillatory potentiation for autaptic weights

**DOI:** 10.3389/fncir.2025.1646317

**Published:** 2025-08-25

**Authors:** Risa Onda, Mihoko Ishida, Kouhei Hattori, Hideaki Yamamoto, Takashi Tanii

**Affiliations:** ^1^Faculty of Science and Engineering, Waseda University, Shinjuku, Tokyo, Japan; ^2^Research Institute of Electrical Communication, Tohoku University, Sendai, Japan

**Keywords:** autapse, STDP, recurrent connection, selection rule, autaptic delay, network-level synchronization

## Abstract

Neuronal networks in animal brains are considered to realize specific filter functions through the precise configuration of synaptic weights, which are autonomously regulated without external supervision. In this study, we employ a single Hodgkin–Huxley-type neuron with autapses as a minimum model to computationally investigate how spike-timing-dependent plasticity (STDP) adjusts synaptic weights through recurrent feedback. The results show that the weights undergo oscillatory potentiation or depression with respect to autaptic delay and high-frequency stimulation. Our findings suggest that the STDP-mediated modulation of autaptic weights, governed by autaptic delay and input frequency, may serve as a mechanism for promoting network-level synchronization in neural systems if the network contains neurons with autapses.

## 1 Introduction

A neuron is believed to act as a filter that integrates input signals and attempts to transmit the signals to the postsynaptic neurons depending on the timing and magnitude of the input. While signals are structured as spike patterns based on the synaptic connectivity, the synaptic weights between neurons change gradually in response to the spike patterns (Hebb, 1949; Bi and Poo, 1998). When postsynapse neurons receive neurotransmitters, intracellular calcium-dependent protein kinases and Rho GTPases are activated, followed by the reorganization of the actin cytoskeleton (Lee et al., 2024, 2009; Murakoshi et al., 2011). This causes a transient increase in spine volume, and the α-amino-3-hydroxy-5-methyl-4-isoxazolepropionic acid (AMPA) receptors inserted into the expanding spine enhance synaptic weight continuously (Lee et al., 2024; Matsuzaki et al., 2001, 2004; Murakoshi and Yasuda, 2011). On the other hand, if the synaptic weight decreases, spike signals are no longer effectively transferred to the postsynaptic neurons; hence, the connection becomes comparatively insignificant. The overall circuit connectivity thus changes dynamically. Such autonomous adjustment undergoes in individual synapses, resulting in a local circuit with a sophisticated connectivity that works as a specific filter.

An autapse is a synaptic structure in which the axon of a neuron forms a connection onto its own dendrites and has been identified in multiple brain regions (Gilson et al., 2009; Loos and Glaser, 1972; Karabelas and Purrura, 1980; Bekkers and Stevens, 1991). For example, in the mammalian cortex, although autapses are approximately one-third as abundant as synapses, they are observed in 80% of the neurons (Lübke et al., 1996) and considered to contribute to temporal tuning, gain control, and network synchronization (Guo et al., 2016; Ma et al., 2015; Protachevicz et al., 2020; Jiang et al., 2012). Thus far, computational simulation has predicted that, if the network contains neurons with autapses, autapses with specific propagation delays can enhance global-network-wide synchronization in the absence of synaptic plasticity (Sillito et al., 1994; Kerr et al., 2013). However, autaptic signals may reenter a presynaptic neuron within 5 milliseconds (Patolsky et al., 2006; Uchino et al., 2022), suggesting that the autaptic weight may be autonomously modulated depending on the neuron's spiking activity. Understanding how neurons regulate autaptic weights offers insights into the coordination of recurrent connections and the autonomous emergence of synaptic configurations that enable specific filter functions.

Here, we report our computational simulations to investigate how a single neuron with autapses adjusts the autaptic weights through spike-timing-dependent plasticity (STDP) (Bi and Poo, 1998; Markram et al., 1997; Kopysova and Debanne, 1998; Song et al., 2000; Letzkus et al., 2006; Gilson et al., 2009; Feldman, 2012). This neuron model was configured with multiple autapses bearing different transmission delays (Lubenov and Siapas, 2008) and was driven by input spike trains generated from a Poisson process at defined frequencies, mimicking presynaptic activity. All autaptic weights were updated according to the STDP rule. We show that the potentiation and depression of autapic weights depend on the interplay between input frequency and transmission delay, offering a delay- and frequency-dependent selection mechanism. We further discuss how such selection may contribute to the synchronization of neuronal firing.

## 2 Computational model

A Hodgkin–Huxley-type model of a cortical neuron was used to simulate the neuronal dynamics (Hodgkin and Huxley, 1952; Pospischil et al., 2008; Hattori et al., 2020). The time evolution of the membrane potential *V*(*t*) was calculated as


(1) $$\begin{array}{l} C_{\text{m}}\frac{dV}{dt}=-I_{\text{Na}}-I_{\text{Kd}}-I_{\text{M}}-I_{\text{leak}}-I_{\text{syn}}+I_{\text{app}}, \end{array}$$

where *C*_m_ is the specific capacitance of the cell membrane, *V* the membrane potential, *I*_Na_ the sodium current, *I*_Kd_ the potassium current, *I*_M_ the slow non-inactivating potassium current responsible for spike-frequency adaptation, *I*_leak_ the leakage current, *I*_syn_ the synaptic current, and *I*_app_ the current applied additionally to the cell. In the simulation, *C*_m_ was assumed to be 1.0 μF/cm^2^ (Pospischil et al., 2008). Numerical integrations were performed using the Euler method with a time step of less than 0.04 ms.

The current terms are given by


(2) $$\begin{array}{l} I_{\text{Na}}={\bar{g}}_{\text{Na}}m^{3}h\left(V-E_{\text{Na}}\right), \end{array}$$

(3) $$\begin{array}{l} I_{\text{Kd}}={\bar{g}}_{\text{Kd}}n^{4}\left(V-E_{\text{K}}\right), \end{array}$$

(4) $$\begin{array}{l} I_{\text{M}}={\bar{g}}_{\text{M}}p\left(V-E_{\text{K}}\right), \end{array}$$

(5) $$\begin{array}{l} I_{\text{leak}}=g_{\text{leak}}\left(V-E_{\text{leak}}\right), \end{array}$$

where ḡ_Na_, ḡ_Kd_, ḡ_M_, and *g*_leak_ indicate the maximum sodium conductance, the maximum potassium conductance, the maximum slow non-inactivating potassium conductance, and the leakage conductance, respectively. In the simulation, ḡ_Na_, ḡ_Kd_, ḡ_M_, and *g*_leak_ were set to 56 mS/cm^2^, 6 mS/cm^2^, 75 μS/cm^2^, and 20.5 μS/cm^2^, respectively (Pospischil et al., 2008). Moreover, the reversal potential for sodium current *E*_Na_, potassium channels *E*_K_, and leakage channels *E*_leak_ were set to 50, −90, and −70.3 mV, respectively (Pospischil et al., 2008). The functionals *m, h*, and *n* are given by


(6) $$\begin{array}{l} \frac{\text{d}x}{\text{d}t}=\alpha_{x}\left(V\right)\left(1-x\right)-\beta_{x}\left(V\right)x\left(x=m,h,n\right), \end{array}$$

where the voltage-dependent functions α_*x*_(*V*) and β_*x*_(*V*) obey the following equations (Pospischil et al., 2008; Hattori et al., 2020):


(7) $$\begin{array}{l} \alpha_{m}\left(V\right)=\frac{-0.32\left(V+43.2\right)}{\exp\left(-\frac{V+43.2}{4}\right)-1}, \end{array}$$

(8) $$\begin{array}{l} \beta_{m}\left(V\right)=\frac{0.28\left(V+16.2\right)}{\exp\left(\frac{V+16.2}{5}\right)-1}, \end{array}$$

(9) $$\begin{array}{l} \alpha_{h}\left(V\right)=0.128\exp\left(-\frac{V+39.2}{18}\right), \end{array}$$

(10) $$\begin{array}{l} \beta_{h}\left(V\right)=\frac{4}{1+\exp\left(-\frac{V+16.2}{5}\right)}, \end{array}$$

(11) $$\begin{array}{l} \alpha_{n}\left(V\right)=\frac{-0.032\left(V+41.2\right)}{\exp\left(-\frac{V+41.2}{5}\right)-1}, \end{array}$$

(12) $$\begin{array}{l} \beta_{n}\left(V\right)=0.5\exp\left(-\frac{V+46.2}{40}\right). \end{array}$$

The functional *p* is given by the following equations (Pospischil et al., 2008):


(13) $$\begin{array}{l} \frac{\text{d}p}{\text{d}t}=\frac{p_{\infty }\left(V\right)-p}{\tau_{p}\left(V\right)}, \end{array}$$

(14) $$\begin{array}{l} p_{\infty }\left(V\right)=\frac{1}{1+\exp\left[-\left(V+35\right)/10\right]}, \end{array}$$

(15) $$\begin{array}{l} \tau_{p}\left(V\right)=\frac{608}{3.3\exp\left[\left(V+35\right)/20\right]+\exp\left[-\left(V+35\right)/20\right]}. \end{array}$$

The synaptic current *I*_syn_ is the current introduced by AMPA and *N*-methyl-D-aspartate (NMDA) receptors and is given by


(16) $$\begin{array}{l} I_{\text{syn}}(t)=\sum_{i}I_{\text{syn},i}(t)=\sum_{i}\left(I_{\text{AMPA},i}(t)+I_{\text{NMDA},i}(t)\right)\\ \;=\sum_{i}(w_{i}g_{\text{AMPA},i}(V(t-\tau_{\text{delay},\text{i}})-V_{\text{syn}})\\ \;+w_{i}g_{\text{NMDA},i}(V(t-\tau_{\text{delay},\text{i}})-V_{\text{syn}})), \end{array}$$

where *w*_*i*_ and *V*_syn_ indicate the synaptic weight and the synaptic reversal potential, respectively (Hattori et al., 2020; Borges et al., 2017). We assumed *V*_syn_ = 0 mV (Yamamoto et al., 2016). The suffix *i* denotes the *i*-th synaptic connection. In the case of an autapse, the synaptic current *I*_syn, *i*_ was fed back to the neuron after a delay of τ_delay_, corresponding to the time required for an action potential to propagate through the axon and transmitted through the autapse. The AMPA conductance *g*_AMPA, *i*_ and the NMDA conductance *g*_NMDA, *i*_ are given by


(17) $$\begin{array}{l} g_{\text{AMPA},i}=\frac{E_{\text{AMPA},i}}{0.37N}, \end{array}$$

(18) $$\begin{array}{l} g_{\text{NMDA},i}=\frac{E_{\text{NMDA},i}}{2.15N\left(1+\frac{\left[\text{M}{\text{g}}^{2+}\right]}{3.57}\exp\left(-0.062V\right)\right)}, \end{array}$$

(19) $$\begin{array}{l} \frac{dR_{x,i}}{dt}=\frac{I_{x,i}}{\tau_{\text{rec},x}}-U_{\text{SE},x}R_{x,i}\exp\left(-\frac{t-t_{\text{AP}}}{\tau_{\text{rise},x}}\right), \end{array}$$

(20) $$\begin{array}{l} \frac{dE_{x,i}}{dt}=-\frac{E_{x,i}}{\tau_{\text{inact},x}}+U_{\text{SE},x}R_{x,i}\exp\left(-\frac{t-t_{\text{AP}}}{\tau_{\text{rise},x}}\right), \end{array}$$

(21) $$\begin{array}{l} I_{x,i}=1-R_{x,i}-E_{x,i}\left(x=\text{AMPA},\text{NMDA}\right), \end{array}$$

where *N* indicates the number of synaptic connections, and *t*_AP_ indicates the time the neuron fires. We assumed τ_rec, AMPA_ = 200ms, τ_inact, AMPA_ = 5ms, *U*_SE, AMPA_ = 0.7, τ_rec, NMDA_ = 200ms, τ_inact, NMDA_ = 55ms, *U*_SE, NMDA_ = 0.03, *V*_syn_ = 0mV, and [Mg^2+^] = 1.0mM (Hattori et al., 2020).

The synaptic (autaptic) weight *w*_*i*_ is updated according to the following equations representing STDP (Borges et al., 2017):


(22) $$\begin{array}{l} w_{i}=w_{i}+\eta \Delta w_{i}, \end{array}$$

(23) $$\Delta w_{i}\;=\{\begin{array}{cc} A_{1}\exp\left(-\frac{\Delta t}{\tau_{1}}\right) & \left(\Delta t\geq 0\right)\\ -A_{2}\exp\left(\frac{\Delta t}{\tau_{2}}\right) & \left(\Delta t<0\right) \end{array},$$

where Δ*t* represents the time difference between the firing of the postsynaptic neuron and the arrival time of a synaptic current from the presynaptic neuron, namely, Δ*t* = *t*_AP_−*t*_EPSC_. Hence, Δ*t* takes both positive and negative values. In the case of an autapse, the pre- and postsynaptic neurons are identical. We assumed that *A*_1_ = 1.0, *A*_2_ = 0.5, τ_1_ = 1.8ms, and τ_2_ = 6.0ms (Borges et al., 2017). We also assumed that *w*_*i*_ is bound to a maximum of one and a minimum of zero. The learning rate η was set to 10^−3^. All autaptic weights were initialized to 0.5 before stimulation and limited to the range of 0–1.

The applied current *I*_app_ models synaptic inputs from other neurons (Dayan and Abbott, 2005; Miller, 2018) and is defined as


(24) $$\begin{array}{l} I_{\text{app}}\left(t\right)=P_{\text{app}}\times \left(\exp\left(-\frac{t-t_{\text{input}}}{\tau_{\text{fall}}}\right)-\exp\left(-\frac{t-t_{\text{input}}}{\tau_{\text{rise}}}\right)\right)\\ \;\times \left(V_{\text{syn}}-V\right), \end{array}$$

where *t*_input_ denotes the onset time of an input event, drawn from an exponential distribution, and *P*_app_ is the transmission intensity of the input current and is set to 10^−2^. The time constants were set to τ_rise_ = 0.2ms and τ_fall_ = 5.3ms (Yamamoto et al., 2016). The synaptic inputs were generated as a Poisson process with a specific mean frequency. The input spike frequency was changed from 200 spikes/s to 5000 spikes/s. For each condition, simulations were repeated 50 times using independently generated inputs, and the results were averaged. The fixed parameters used in the present simulation are summarized in Supplementary Table 1.

## 3 Results and discussion

**Figure 1:**
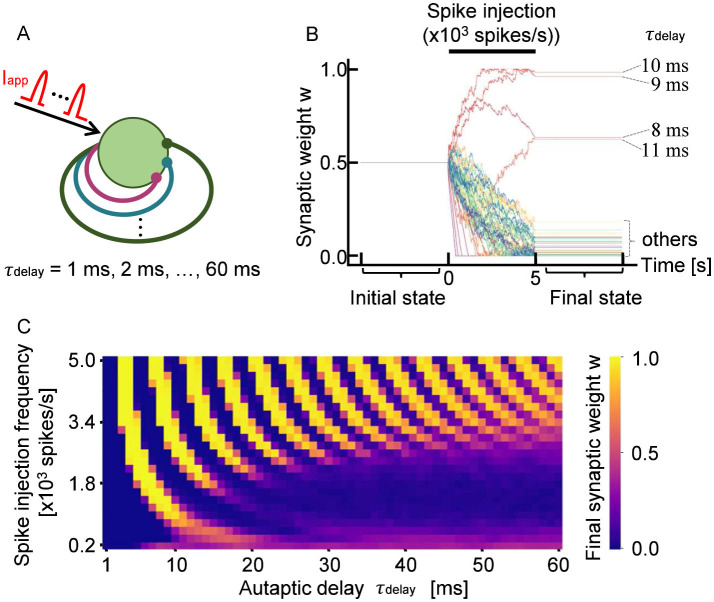
**(A)** Schematic of a neuron with 60 autapses, each with a distinct propagation delay, τ_delay_. The neuron receives stochastic input spikes *I*_app_ with a mean frequency of 10^3^ spikes per second. **(B)** Representative time evolution of synaptic weights *w* during 5 s of stimulation. Each trace represents an individual weight that was updated in response to neuronal firing. The autaptic delay τ_delay_ is annotated for autapses that were potentiated. **(C)** Final synaptic weights of autapses. The observed stripe pattern indicates that STDP potentiates or depresses autapses depending on their propagation delay.

We first investigated how the synaptic weights of STDP-regulated autapses are modulated when a neuron receives external spike inputs. The simulation model is illustrated in Figure 1A. A single neuron with 60 autapses, each with a unique autaptic delay (1–60 ms), was simulated. A spike train *I*_app_, modeling input spikes from other neurons, was applied for 5 s, and the time evolution of synaptic weights of autapses was analyzed.

Figure 1B shows a representative example of how the synaptic weights of STDP-regulated autapses evolve in response to external inputs. The synaptic weight set to be 0.5 at the starting point was updated during the spike injection. Minor fluctuations observed in individual synaptic weights originated from the variability in spike timing caused by stochasticity in *I*_app_. Notably, it was found that autapses with specific delays tended to be selectively potentiated when the input frequency was fixed. This property remained consistent across multiple simulations using different random seeds for generating *I*_app_. As shown in Figure 1B, autapses with delays of approximately 10 ms (8 ms < τ_delay_ < 12 ms) were robustly potentiated by input stimulation with a frequency of 10^3^ spikes per second. In contrast, autapses with shorter (τ_delay_ < 8 ms) or longer (τ_delay_>12 ms) delays tended to be depressed under the same stimulation condition.

A similar trend was also observed when input stimulation was delivered at other frequencies. Specifically, the evolution of the weights could be classified into three categories: (i) those that gradually potentiated and reached saturation, (ii) those that gradually depressed and stabilized at low values, and (iii) those that fluctuated within an intermediate range (τ_delay_ = 8 ms and τ_delay_ = 11 ms). Figure 1C shows the final synaptic weights for each autaptic delay and input frequency, with each of the three categories represented with different colors. The simulation was performed fifty times with different spike trains, and the average eventual synaptic weight was taken. As shown in Figure 1C, the eventual synaptic weight shows a periodicity with alternating patterns of potentiation and depression depending on the autaptic delay. Thus, STDP modulates the weights of autapses in a delay-dependent manner, enabling the specific selection of autaptic connections.

**Figure 2:**
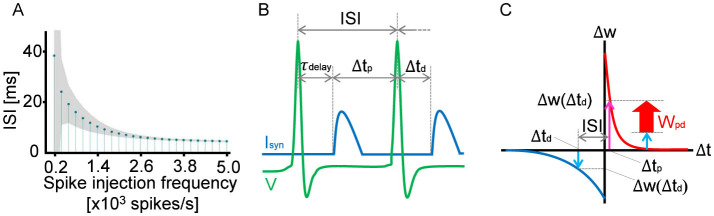
**(A)** Relationship between the average ISI of a neuron with 60 autapses and the average spike injection frequency. The shaded area of the lollipop chart indicates the standard deviation. **(B)** Schematic showing how to determine Δ*t*_p_ and Δ*t*_d_ for the given ISI and autaptic delay. Δ*t*_p_ is defined as the duration from the arrival time of the former feedback synaptic current *I*_syn_ to the subject spike (the second spike in the figure), whereas Δ*t*_d_ is defined as the duration from the spike to the arrival time of the latter synaptic current. In the case of τ_delay_ ≤ ISI, Δ*t*_d_ is equivalent to −τ_delay_ and Δ*t*_p_ is equivalent to ISI−τ_delay_, as shown in the schematic. In the case of τ_delay_ > ISI, Δ*t*_d_ and Δ*t*_p_ can be determined accordingly. The potentiation and depression of the synaptic weight can be estimated using the STDP curve, as shown in **(C)**. At every spike timing, the synaptic weight is updated in accordance with the balance between Δ*w* (Δ*t*_p_) and Δ*w* (Δ*t*_d_). If Δ*w*(Δ*t*_p_) > Δ*w*(Δ*t*_d_), the synaptic weight is potentiated. Otherwise, the synaptic weight is depressed.

In STDP, spike timing is the key to connection strength modulation. Therefore, the interspike interval (ISI) is an essential indicator when discussing changes in neuron connection strength.Figure 2A shows the relationship between the ISI and the spike injection frequency. The ISI is long at a low frequency and asymptotically approaches the minimum value as the spike injection frequency increases. The minimum value is attributed to the refractory period of the neuron. In the following analyses, we use the ISI rather than the spike injection frequency because the ISI can be compared directly with the autaptic delay on the temporal axis, as shown in Figure 2B.

Every spike injection updates synaptic weights, and it is the STDP curve that determines whether the synaptic weight is potentiated or depressed. In the case of autapses, input signals are assumed to be feedback signals via the autaptic connection. As shown in Figure 2B, the feedback synaptic current *I*_syn_ arrives with the autaptic delay τ_delay_ after the first spike, depolarizing the membrane potential. Therefore, Δ*t*_p_ is defined as the duration from the arrival time of *I*_syn_ to the second spike. Δ*t*_d_ is also defined as the duration from the second spike to the subsequent arrival time of *I*_syn_, which is equivalent to τ_delay_ in the case of ISI>τ_delay_. As shown in Figure 2C, the potentiation component Δ*w*(Δ*t*_p_) was evaluated using the STDP curve. Similarly, the depression component Δ*w*(Δ*t*_d_) was estimated as a negative value. Since these potentiation and depression components are competing in STDP, the synaptic weight must be updated depending on the balance between Δ*w*(Δ*t*_p_) and Δ*w*(Δ*t*_d_); namely, *W*_pd_ = Δ*w*(Δ*t*_p_)+Δ*w*(Δ*t*_d_). As a result, if τ_delay_ is close to the ISI but does not exceed the ISI, the synaptic weight of the autapse is potentiated. This is because the neuron fires immediately after *I*_syn_ arrives. In contrast, if τ_delay_ exceeds the ISI but is close to the ISI, the synaptic weight of the autapse is depressed because the neuron fires immediately before *I*_syn_ arrives.

As shown in Figure 2C, *W*_pd_ is constant at a constant ISI. This condition is fulfilled at a high spike injection frequency because, as shown in Figure 2A, the fluctuation in ISI decreases with increasing spike injection frequency. Therefore, as shown in Figure 1C, the oscillation in the autapse selection becomes clear in the high-frequency region. In contrastingly, depression is dominant at a low frequency because of the asymmetric characteristics in the potentiation and depression of the STDP curve.

The STDP autapses undergo selections according to the autaptic delay, particularly at a high spike injection frequency, as shown in Figure 1C. Although the selection was made only on the local autapses, it was reported that a single autapse may induce network synchronization. Yilmaz et al. (2016) simulated a neuronal network containing a single autapse and reported that network synchronization occurs depending on the autaptic delay and conductance. Moreover, Wang et al. (2015) reported that the autaptic delay inducing network synchronization resulted in discrete values. Similar results were obtained by the simulation of a more complicated network. Note that the autaptic delay and conductance were controlled artificially in the simulation reported previously, where neuronal plasticity was not modeled. In this work, neuronal plasticity was realized in the presented simulation, where the selection was made spontaneously in a spike-timing-dependent manner. The above results suggest that the selection of autapses regulates the network activity such as network synchronization through neuronal plasticity.

It was reported in Graupner and Brunel (2012) that, in physiological calcium concentrations, not the STDP function like the one shown in Figure 2C but the depression-potentiation-depression (DPD) function is realistic. Thus, the final synaptic weights of autapses were calculated by using the DPD function proposed in Graupner and Brunel (2012). Supplementary Figure 1 shows that the autaptic weights undergo oscillatory potentiation or depression with respect to autaptic delay and high-frequency stimulation. This is because the balance between potentiation and depression changes periodically with increasing autaptic delay. The small discrepancy between Figure 1C and Supplementary Figure 1 is due to the difference of the STDP curve used in the present simulations.

On the other hand, it was reported that the STDP function like the one shown in Figure 2C is usually unapplicable in physiological calcium conditions (Inglebert et al., 2020) but is applicable only when the postsynaptic neuron fires at a high frequency (>10 Hz). As shown in Figure 2A, the average ISI of the neuron with sixty autapses is shorter than 40 ms, which corresponds to the average firing frequency higher than 25 Hz. Hence, it is conjectured that the STDP function like the one shown in Figure 2C is applicable to the neuron with sixty autapses.

The simulation reported previously (Borges et al., 2017) employed η = 10^−3^ as the coefficient to update synaptic weights in the simulation. In this work, the same coefficient was used accordingly. However, it is suggested that a smaller coefficient (η≃10^−6^) is realistic because the changes in synaptic weight that real neurons undergo in response to a single input are more gradual (Bi and Poo, 1998). The decrease in η by three orders of magnitude may retard the change in autaptic weight markedly. Nevertheless, if we assume a longer spike injection duration (e.g., 2 h), the autaptic weights are conjectured to converge into the same values, suggesting that the present simulation can be considered an accelerated test.

**Figure 3:**
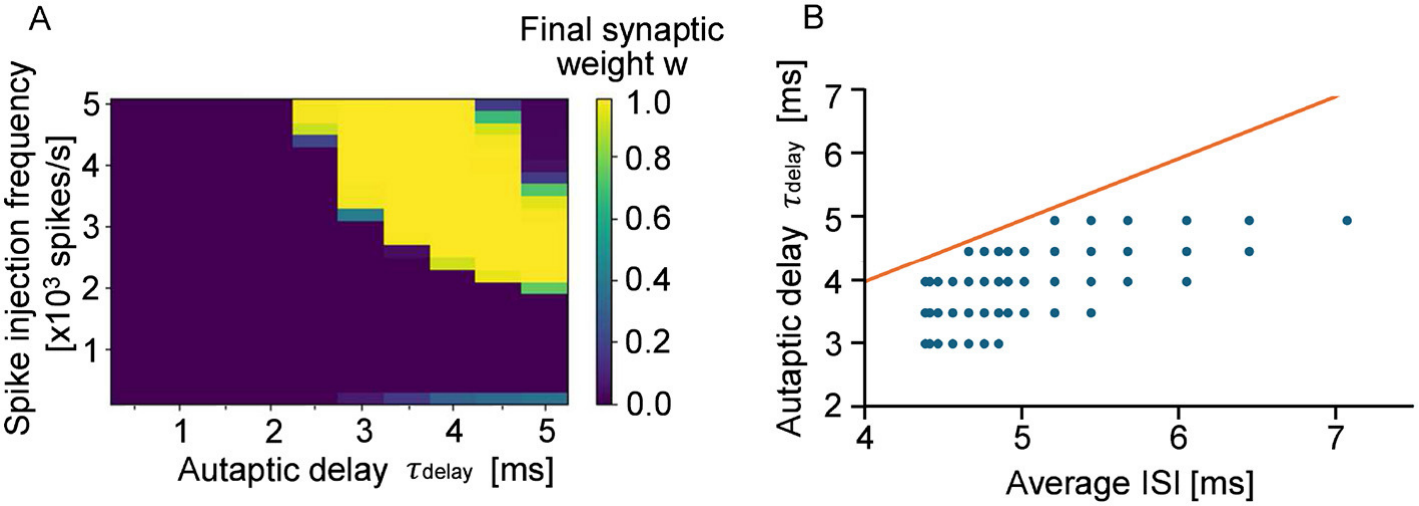
**(A)** Eventual synaptic weight of autapses with autaptic delays of 0.5–5 ms. The bright pixels show potentiated autapses, whereas the dark pixels show depressed autapses. **(B)** Relationship between ISI and autaptic delay. The red line shows that the ISI and the autaptic delay are equivalent. The blue dots show the autapses whose synaptic weights are potentiated and almost saturated in the stimmulation.

As shown in Figure 1C, clear periodic differences in connection strength were observed by assuming autapses with a wide range of propagation delays. However, in the case of cultured neurons and neurons in vivo, autapses with a propagation delay time of 60 ms are not realistic because the time scale is very long. Given that the signal conduction velocity along dendrites is approximately 0.16 m/s and that the average dendritic length of neurons with autapses is about 330 μm, the corresponding autaptic delay is estimated to be 5 ms or less (Patolsky et al., 2006; Uchino et al., 2022). In Figure 3A, we show how the final synaptic weights of STDP-regulated autapses with delays ranging from 0.5 to 5 ms as a function of input stimulation frequency. Clearly, synaptic weights were either potentiated or depressed depending on the autaptic delay. Autapses with delays shorter than 2 ms were consistently depressed because of the refractory period of a neuron (Yeomans, 1978), suggesting that short-delay autapses are insignificant. Even among autapses with delays exceeding 2 ms, synaptic depression was dominant when the input signal frequency was below 2,000 spikes per second. Although this simulation did not include autapses with delays longer than 5 ms, the results within this range closely align with those presented in Figure 1C. This indicates that the effects of individual autapses on the potentiation/depression of synaptic weights are independent. Figure 3B further reveals that the autapses with delays slightly shorter than the average ISI are preferentially potentiated. These findings suggest that the autapses with delays between 2 and 5 ms are most effective in promoting network synchronization if it is induced by autaptic feedback.

As reported in Boudkkazi et al. (2007) and Boudkkazi et al. (2011), the synaptic delay increases with decreasing presynaptic release probability by 1–2 ms. The synaptic delay also increases with increasing duration and amplitude of the presynaptic action potential by 1–2 ms. If these conditions are applied for the presented simulations, the autaptic delay corresponding to the horizontal axis of Figures 1C, 3A and Supplementary Figure 1 may shift by 1–2 ms accordingly.

## 4 Conclusions

In this study, we analyzed how STDP modulates the synaptic weights of a single neuron with autapses by computational simulation. We found that synaptic weights of autapses exhibit oscillatory patterns of potentiation and depression depending on the autaptic delay. The oscillation was also found to vary with the frequency of input stimulation. In particular, focusing on the physiologically realistic range of autaptic delays (τ_delay_ < 5 ms), our results indicate that autapses with a specific delay are selectively potentiated under high-frequency inputs, suggesting their role in regulating network dynamics such as network synchronization. Taken together, STDP modulates the synaptic weights of autapses in a delay-dependent manner, enabling the specific selection of autaptic connections depending on the input spike frequency.

## Data Availability

The original contributions presented in the study are included in the article/Supplementary material, further inquiries can be directed to the corresponding author.
