# Supplementary material for: Spike-timing-dependent plasticity offers delay-gated oscillatory potentiation for autaptic weights
Source: Front Neural Circuits. 2025 Aug 25;19:1646317. doi: 10.3389/fncir.2025.1646317 (PMC12414951; doi:10.3389/fncir.2025.1646317)
Supplement: Supplementary file 1 [file Presentation_1.pdf]

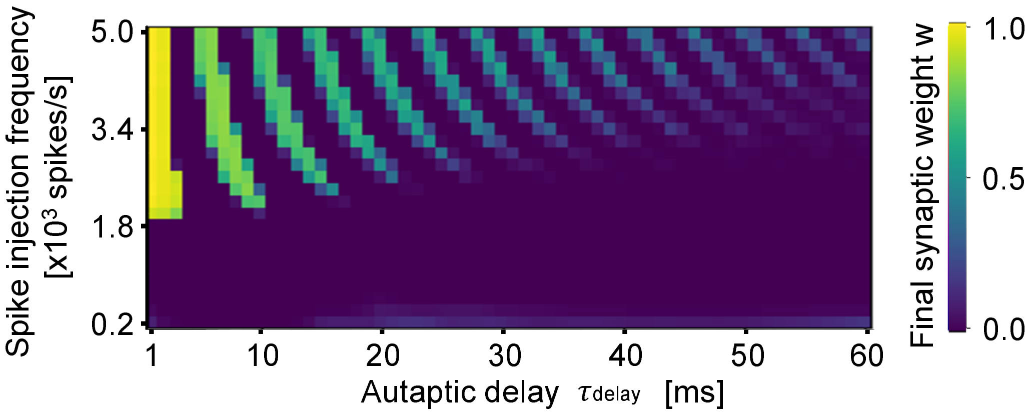


**Supplementary Figure 1.** Final synaptic weights of autapses calculated by using depression-potentiation-depression (DPD) curve introduced in Graupner and Brunel (2012). The observed stripe pattern indicates that STDP potentiates or depresses autapses depending on their propagation delay.

**Supplementary Table 1.** Fixed parameters used in the simulation.

| Parameter | Description | Value | Equation |
|---|---|---|---|
| $C_\mathrm{m}$ | The specific capacitance of the cell membrane | 1.0 [$\mu$F/cm$^2$] | (1) |
| $dt$ | A time step | 0.04 [ms] for Fig. 1<br>0.025 [ms] for Fig. 3 | (1)(6)(13)<br>(19)(20) |
| $\bar{g}_\mathrm{Na}$ | The maximum sodium conductance | 56 [mS/cm$^2$] | (2) |
| $\bar{g}_\mathrm{Kd}$ | The maximum potassium conductance | 6 [mS/cm$^2$] | (3) |
| $\bar{g}_\mathrm{M}$ | The maximum slow non-inactivating potassium conductance | 75 [$\mu$S/cm$^2$] | (4) |
| $g_\mathrm{leak}$ | The leakage conductance | 20.5 [$\mu$S/cm$^2$] | (5) |
| $E_\mathrm{Na}$ | The reversal potential for sodium current | 50 [mV] | (2) |
| $E_\mathrm{K}$ | The reversal potential for potassium channels | -90 [mV] | (3)(4) |
| $E_\mathrm{leak}$ | The reversal potential for leakage channels | -70.3 [mV] | (5) |
| $V_\mathrm{syn}$ | The synaptic reversal potential | 0 [mV] | (16) |
| $\tau_\mathrm{rec,AMPA}$ | The time constant for recovery of AMPA receptors | 200 [ms] | (20) |
| $\tau_\mathrm{inact,AMPA}$ | The time constant for inactivation of AMPA receptors | 5 [ms] | (19) |
| $\tau_\mathrm{rise,AMPA}$ | The time constant for the rise of AMPA receptors | 1.1 [ms] | (19)(20) |
| $U_\mathrm{SE,AMPA}$ | The glutamate release probability of AMPA receptors | 0.7 | (19)(20) |
| $\tau_\mathrm{rec,NMDA}$ | The time constant for recovery of NMDA receptors | 200 [ms] | (20) |
| $\tau_\mathrm{inact,NMDA}$ | The time constant for inactivation of NMDA receptors | 55 [ms] | (19) |
| $\tau_\mathrm{rise,NMDA}$ | The time constant for the rise of NMDA receptors | 145 [ms] | (19)(20) |
| $U_\mathrm{SE,NMDA}$ | The glutamate release probability of NMDA receptors | 0.03 | (19)(20) |
| [$Mg^{2+}$] | The extracellular magnesium ion concentration | 1.0 [mM] | (18) |
| $A_1$ | The magnitude for the potentiation region of the STDP function | 1.0 | (23) |
| $A_2$ | The magnitude for the depression region of the STDP function | 0.5 | (23) |
| $\tau_1$ | The time constant for the potentiation region of the STDP function | 1.8 [ms] | (23) |
| $\tau_2$ | The time constant for the depression region of the STDP function | 6.0 [ms] | (23) |
| $\eta$ | The learning rate of the STDP function | $10^{-3}$ | (22) |
| $P_\mathrm{app}$ | The transmission intensity of the input current | $10^{-2}$ | (24) |
| $\tau_\mathrm{rise}$ | The time constant for the rise of injection current | 0.2 [ms] | (24) |
| $\tau_\mathrm{fall}$ | The time constant for the fall of injection current | 5.3 [ms] | (24) |
